# Supplementary material for: Feeling heard: Operationalizing a key concept for social relations
Source: PLoS One. 2023 Nov 30;18(11):e0292865. doi: 10.1371/journal.pone.0292865 (PMC10688667; doi:10.1371/journal.pone.0292865)
Supplement: S1 Appendix — (DOCX) [file pone.0292865.s001.docx]

**Appendix**

**Table of Contents**

| **1.1 Tables & figure Study 1** | **2** |
| --- | --- |
| **1.2 Questionnaire Study 1** | **10** |
| **1.3 Interviews Study 1** | **18** |
| **2.1 Tables & figure Study 2** | **22** |
| **2.2 Questionnaire Study 2** | **27** |
| **2.3 Reasoning behind the scales included to assess validity** | **40** |
| **3.1 Study 3** | **42** |
| **3.2 Questionnaire Study 3** | **50** |
| **4 The Feeling Heard Scale user manual** | **56** |

**1.1 Tables & figure** **Study 1**

**Table 1. A characterization of the described conversation based on the percentage of participants scoring in each category of the conversation characteristics.**

| Question | Answer option | Part A  (*N* = 194)^1^ | Part B  (*N* = 214)^1^ |
| --- | --- | --- | --- |
| Good internet-connection? | Yes | 90.21% | 93.93% |
|  | A bit | 7.73% | 5.14% |
|  | No | 2.06% | 0.93% |
| How many interaction partner(s)? | One | 57.22% | 57.94% |
|  | Two | 8.25% | 10.28% |
|  | Three | 5.67% | 8.88% |
|  | More than three | 28.87% | 22.90% |
| Acquaintance before the conversation? | Yes | 61.34% | 68.69% |
|  | A bit | 8.76% | 8.41% |
|  | No | 29.90% | 22.90% |
| Good relationship? | Yes | 50.00% | 67.29% |
|  | A bit | 26.80% | 18.69% |
|  | No | 23.20% | 14.02% |
| Equal relationship? | Equal | 62.89% | 74.77% |
|  | Self authority | 4.12% | 5.14% |
|  | Other authority | 22.68% | 14.95% |
|  | Don’t know | 10.31% | 5.14% |

*Note.* ^1^ *N*’s after excluding the non-serious conversation descriptions. Percentages are calculated relative to these.

**Table 2. The means, standard deviations, skewness, and kurtosis of the original set of 16 feeling heard items. The last column shows the result of the t-test of the differences between (feeling heard and feeling not heard) conditions, with the felt heard conversation as the reference condition.**

| Item | Mean (SD) | Skewness | Kurtosis | t (df) |
| --- | --- | --- | --- | --- |
| 1. I Felt Heard | 3.47 (1.44) | -0.50 | 1.67 | 23.12 (124.84) |
| 2. I Speak Freely | 3.82 (1.04) | -0.89 | 3.09 | 9.75 (117.56) |
| 3. I Speak Mind | 3.60 (1.12) | -0.43 | 2.18 | 11.39 (142.58) |
| 4. I Felt Inhibited | 2.42 (1.22) | 0.58 | 2.16 | -6.17 (165.83) |
| 5. Other Self Concerned | 2.68 (1.36) | 0.30 | 1.78 | -15.07 (182.03) |
| 6. Other Listened | 3.53 (1.20) | -0.59 | 2.36 | 15.11 (130.01) |
| 7. Other Attentive | 3.59 (1.21) | -0.65 | 2.52 | 15.16 (123.88) |
| 8. Other Took Perspective | 3.27 (1.20) | -0.36 | 2.21 | 14.83 (148.80) |
| 9. Other Insensitive | 2.58 (1.26) | 0.40 | 2.07 | -11.74 (173.11) |
| 10. Other Emphatic | 3.21 (1.08) | -0.25 | 2.54 | 7.90 (188.42) |
| 11. Other Genuine Interest | 3.40 (1.18) | -0.45 | 2.29 | 16.00 (145.78) |
| 12. Other Took Serious | 3.61 (1.17) | -0.68 | 2.57 | 15.65 (119.39) |
| 13. Other Respectful | 3.72 (1.11) | -0.88 | 3.21 | 12.19 (118.26) |
| 14. We Different Perspective | 3.20 (1.18) | -0.13 | 2.13 | -6.62 (186.29) |
| 15. We Understood | 3.34 (1.21) | -0.50 | 2.17 | 17.21 (133.17) |
| 16. We Wavelength | 3.26 (1.21) | -0.39 | 2.07 | 17.37 (141.63) |

*Note.* ^a^ Welch two-sample to-sided t-test of the difference between conditions (feeling heard vs not feeling heard). All were significant at *** *p* < .001.

**Table 3.** **The Pearson correlations between the 16 feeling heard items.**

|  | 2 | 3 | 4 | 5 | 6 | 7 | 8 | 9 | 10 | 11 | 12 | 13 | 14 | 15 | 16 |
| --- | --- | --- | --- | --- | --- | --- | --- | --- | --- | --- | --- | --- | --- | --- | --- |
| 1 | .57*** | .65*** | -.34*** | -.76*** | .81*** | .79*** | .81*** | -.71*** | .51*** | .83*** | .83*** | .78*** | -.46*** | .86*** | .87*** |
| 2 |  | .81*** | -.58*** | -.61*** | .64*** | .66*** | .52*** | -.47*** | .37*** | .55*** | .57*** | .53*** | -.15* | .55*** | .57*** |
| 3 |  |  | -.51*** | -.66*** | .72*** | .73*** | .66*** | -.54*** | .48*** | .64*** | .60*** | .60*** | -.21** | .62*** | .64*** |
| 4 |  |  |  | .55*** | -.35*** | -.33*** | -.27*** | .39*** | -.20*** | -.30*** | -.35*** | -.31*** | .20** | -.40*** | -.36*** |
| 5 |  |  |  |  | -.78*** | -.76*** | -.69*** | .76*** | -.48*** | -.73*** | -.69*** | -.65*** | .41*** | -.74*** | -.73*** |
| 6 |  |  |  |  |  | .90*** | .79*** | -.6***7 | .50*** | .82*** | .80*** | .76*** | -.35*** | .78*** | .78*** |
| 7 |  |  |  |  |  |  | .80*** | -.65*** | .50*** | .84*** | .76*** | .78*** | -.32*** | .74*** | .74*** |
| 8 |  |  |  |  |  |  |  | -.65*** | .55*** | .86*** | .80*** | .80*** | -.40*** | .79*** | .81*** |
| 9 |  |  |  |  |  |  |  |  | -.37*** | -.68*** | -.64*** | -.60*** | .46*** | -.71*** | -.67*** |
| 10 |  |  |  |  |  |  |  |  |  | .53*** | .49*** | .46*** | -.28*** | .50*** | .51*** |
| 11 |  |  |  |  |  |  |  |  |  |  | .82*** | .82*** | -.36*** | .80*** | .80*** |
| 12 |  |  |  |  |  |  |  |  |  |  |  | .87*** | -.37*** | .81*** | .83*** |
| 13 |  |  |  |  |  |  |  |  |  |  |  |  | -.35*** | .76*** | .77*** |
| 14 |  |  |  |  |  |  |  |  |  |  |  |  |  | -.49*** | -.48*** |
| 15 |  |  |  |  |  |  |  |  |  |  |  |  |  |  | .92*** |

*Note.* **p* < .05; ***p* < .01; ****p* < .001.

**Table 4. Modification indices > 10 of the hypothesized model.**

| Parameter | Modification indices | Expected parameter change |
| --- | --- | --- |
| 4 ~~ 5 | 40.94 | .37 |
| 5 ~~ 9 | 34.16 | .29 |
| 13 ~~ 12 | 29.10 | .12 |
| 11 ~~ 12 | 16.95 | -.10 |
| 3 ~~ 8 | 14.76 | .10 |
| 2 ~~ 4 | 12.51 | -.17 |
| Common ground =~5 | 11.82 | -.37 |
| Attention =~11 | 11.78 | .42 |
| 7 ~~ 12 | 10.12 | -.06 |

*Note.* ~~ indicates a covariance. =~ indicates a factor loading.

**Table 5. Residual correlation matrix of the CFA on the hypothesized model. The item numbering is different from the one specified elsewhere because of the item order in the five latent factors.**

|  | 2 | 3 | 4 | 5 | 6 | 7 | 8 | 9 | 10 | 11 | 12 | 13 | 14 | 15 | 16 |
| --- | --- | --- | --- | --- | --- | --- | --- | --- | --- | --- | --- | --- | --- | --- | --- |
| 1. I Felt Heard | -.02 | .01 | .05 | .00 | -.05 | -.02 | .00 | -.01 | -.03 | -.03 | .00 | .02 | .00 | .00 | .00 |
| 2. I Speak Freely |  | .00 | -.08 | -.03 | -.02 | .00 | .00 | -.07 | .02 | -.02 | -.02 | .00 | -.02 | .15 | -.01 |
| 3. I Speak Mind |  |  | .02 | .00 | -.04 | .01 | .08 | .03 | -.01 | .01 | .03 | -.01 | .00 | .12 | .02 |
| 4. I Felt Inhibited |  |  |  | .09 | .17 | .10 | .04 | .11 | .07 | .05 | .07 | .02 | -.02 | .01 | .02 |
| 5. Other Listened |  |  |  |  | .00 | .01 | .00 | -.02 | -.01 | -.02 | .02 | .00 | .01 | .05 | .01 |
| 6. Other Self Con-cerned |  |  |  |  |  | .02 | -.03 | .01 | .18 | .03 | -.03 | .01 | -.07 | .06 | -.06 |
| 7. Other Attentive |  |  |  |  |  |  | .00 | .00 | .01 | .00 | .04 | -.04 | -.02 | .08 | -.03 |
| 8. Other Emphatic |  |  |  |  |  |  |  | .05 | .05 | -.04 | .01 | -.02 | .00 | -.02 | .00 |
| 9. Other Took Perspec-tive |  |  |  |  |  |  |  |  | .01 | .01 | .03 | -.01 | -.02 | .02 | .00 |
| 10. Other Insen-sitive |  |  |  |  |  |  |  |  |  | .05 | .00 | .03 | -.05 | .11 | .00 |
| 11. Other Respect-ful |  |  |  |  |  |  |  |  |  |  | -.01 | .05 | -.02 | .06 | -.01 |
| 12. Other Genuine Interest |  |  |  |  |  |  |  |  |  |  |  | -.03 | .00 | .06 | -.01 |
| 13. Other Took Serious |  |  |  |  |  |  |  |  |  |  |  |  | .02 | .05 | .03 |
| 14. We Under-stood |  |  |  |  |  |  |  |  |  |  |  |  |  | -.01 | .00 |
| 15. We Different Perspec-tive |  |  |  |  |  |  |  |  |  |  |  |  |  |  | .00 |
| 16. We Wave-length |  |  |  |  |  |  |  |  |  |  |  |  |  |  |  |


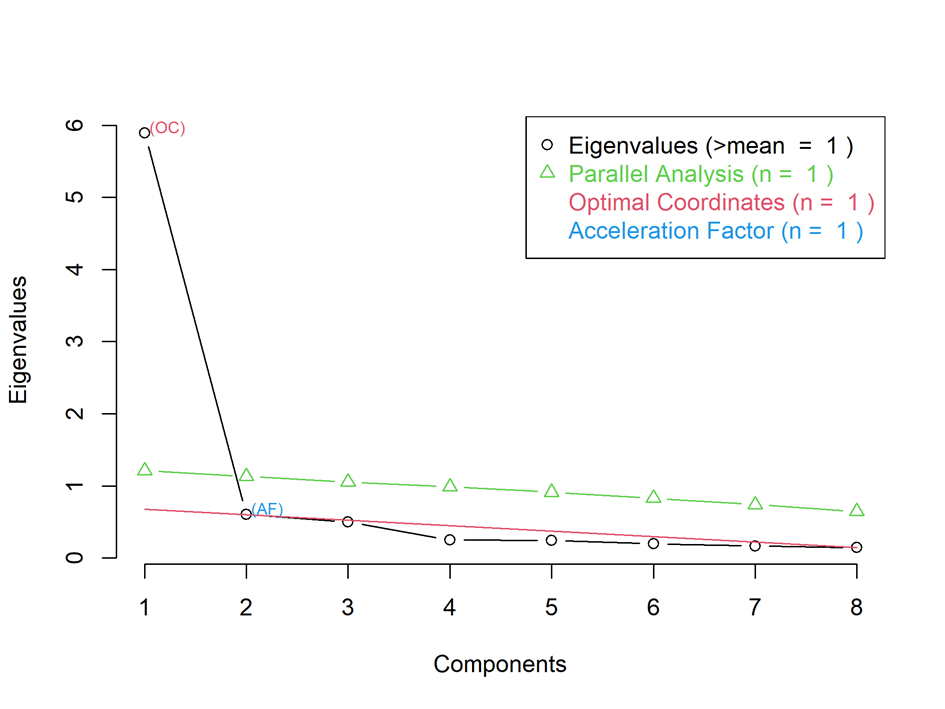


**Figure 1. Scree plot of the eight items.**

**Table 6. Modification Indices > 10 of the CFA with one factor and eight items.**

| Parameter | Modification indices | Expected parameter change |
| --- | --- | --- |
| 5 ~~ 9 | 23.91 | .23 |
| 8 ~~ 13 | 15.71 | .12 |
| 1 ~~ 15 | 10.96 | .10 |

*Note.* ~~ indicates a covariance.

**Table 7. Residual correlation matrix of the CFA with one factor and eight items.**

| Item | 2 | 5 | 6 | 8 | 9 | 13 | 15 |
| --- | --- | --- | --- | --- | --- | --- | --- |
| 1 | -.02 | .01 | -.01 | .00 | .01 | .00 | .03 |
| 2 |  | -.07 | .07 | -.04 | .03 | -.01 | -.03 |
| 5 |  |  | -.04 | .04 | .11 | .06 | .01 |
| 6 |  |  |  | .01 | .02 | .01 | -.03 |
| 8 |  |  |  |  | .02 | .06 | .00 |
| 9 |  |  |  |  |  | .05 | -.01 |
| 13 |  |  |  |  |  |  | .00 |

**Table 8. Modification Indices > 10 of the CFA of the final model.**

| Parameter | Modification indices | Expected parameter change |
| --- | --- | --- |
| 8 ~~ 13 | 13.52 | .11 |
| 5 ~~ 6 | 12.66 | -.12 |
| 1 ~~ 15 | 10.25 | .10 |

*Note.* ~~ indicates a covariance.

**Table 9. Residual correlation matrix of the CFA of the final model.**

| Item | 2 | 5 | 6 | 8 | 9 | 13 | 15 |
| --- | --- | --- | --- | --- | --- | --- | --- |
| 1 | -.02 | .00 | -.01 | -.01 | -.01 | -.01 | .02 |
| 2 |  | -.08 | .07 | -.04 | .01 | -.01 | -.03 |
| 5 |  |  | -.05 | .04 | .00 | .05 | .00 |
| 6 |  |  |  | .01 | .00 | .00 | -.03 |
| 8 |  |  |  |  | .01 | .06 | -.01 |
| 9 |  |  |  |  |  | .04 | -.03 |
| 13 |  |  |  |  |  |  | -.01 |

**Table 10. Results of the measurement invariance test on the data of Part B.**

| Model | χ2 (df) | CFI | RMSEA | Model comp. | Δχ2 (Δdf) | ΔCFI | ΔRMSEA |
| --- | --- | --- | --- | --- | --- | --- | --- |
| M1 configural invariance | 75.04 (38) | .98 | .09 |  |  |  |  |
| M2 metric invariance | 94.75 (45) | .97 | .09 | M1 | 19.77 (7)** | .007 | .006 |
| M3 Scalar invariance | 115.52 (52) | .97 | .09 | M2 | 24.21 (7)** | .006 | .002 |
| M4 Residual invariance | 131.29 (53) | .96 | .10 | M3 | 34.14 (1)*** | .007 | .009 |

*Note.* *N* total = 384; *N* group 1 = 192; *N* group 2 = 192.

Method: maximum likelihood estimation with robust standard errors and a Satorra-Bentler scaled test statistic.

***p* ≤ .01; ****p* ≤ .001.

**Table 11. The means, standard deviations, and inter-item correlations of the eight items of the feeling heard scale in Part B.**

| Item | Mean (SD) | 2 | 3 | 4 | 5 | 6 | 7 | 8 |
| --- | --- | --- | --- | --- | --- | --- | --- | --- |
| 1 | 4.07 (1.10) | .66 | -.56 | .66 | .64 | -.43 | .74 | .70 |
| 2 | 4.10 (0.95) |  | -.52 | .68 | .59 | -.46 | .69 | .68 |
| 5 | 2.21 (1.14) |  |  | -.57 | -.47 | .69 | -.56 | -.54 |
| 6 | 3.94 (0.99) |  |  |  | .63 | -.42 | .70 | .72 |
| 8 | 3.55 (0.99) |  |  |  |  | -.46 | .65 | .65 |
| 9 | 2.16 (1.09) |  |  |  |  |  | -.55 | -.56 |
| 13 | 4.08 (0.93) |  |  |  |  |  |  | .84 |
| 15 | 3.98 (0.94) |  |  |  |  |  |  |  |

*Note.* All correlations were significant at *p* < .001.

**1.2 Questionnaire Study 1^[[1]](#footnote-1),^^[[2]](#footnote-2)^**

To participate in this study, you need to be 18 years or older. Is this the case for you?

- Yes
- No

In addition, you must have experience with conducting online conversations. Is this the case for you?

- Yes
- No

Finally, Dutch should be your native language. Is this the case for you?

- Yes
- No

First of all, we would like to ask you some general questions.

What is your age?

- 18-24 years
- 25-31 years
- 32-38 years
- 39-45 years
- 46-52 years
- 53-59 years
- 60-67 years
- 67+ years

What is your gender?

- Male
- Female
- Other, namely: ______

What is your origin?

- The Netherlands
- Other, namely: ______

In which province do you live?

- Limburg
- Noord-Holland
- Zuid-Holland
- Utrecht
- Overijssel
- Drenthe
- Groningen
- Friesland
- Noord-Brabant
- Flevoland
- Gelderland

What is your current living status?

- Single
- Single with child/children
- Co-habiting
- Cohabiting with child/children

Which of the following options describes your current situation best?

- Studying
- Part-time job
- Full-time job
- Volunteerwork
- Unemployed
- Retired
- Incapacitated

What is the highest education level you have completed?^[[3]](#footnote-3)^

- Geen
- Basisschool
- Lager/voorbereidend onderwijs (lbo/vmbo)
- Middelbaar algemeen voortgezet onderwijs (mavo)
- Middelbaar beroepsonderwijs (mbo)
- Hoger algemeen voortgezet onderwijs (havo)
- Voorbereidend wetenschappelijk onderwijs (vwo)
- Hoger beroepsonderwijs (hbo)
- Wetenschappelijk onderwijs (WO)

*Now describe the last ONLINE conversation you had (use at least 100 characters).*

*What was the situation?*^[[4]](#footnote-4)^

________________________________________________________________

________________________________________________________________

*Now describe an ONLINE conversation in which you felt heard (use at least 100 characters).*

*What was the situation?*

________________________________________________________________

________________________________________________________________

*Why did you feel heard?*

________________________________________________________________

________________________________________________________________

*Now describe an ONLINE conversation in which you felt unheard (use at least 100 characters).*

*What was the situation?*
________________________________________________________________

________________________________________________________________

*Why did you feel unheard?*

________________________________________________________________

________________________________________________________________

The following questions will be about your experiences during the online conversation you just described.

Was the internet connection good during this conversation?

- Yes
- A bit
- No

How many other people participated in this conversation?

- 1
- 2
- 3
- More than 3, namely: _____

Did you know this person before the conversation started?

- Yes
- A bit
- No

Do you have a good relationship with this person?

- Yes
- A bit
- No

Do you have an equal relationship with this person?

- Yes, we are equals
- No, the other has more authority (e.g., the other is my employer)
- No, I have more authority (e.g., the other is my children)
- Don’t know

To what extent do you agree with the following statement?

In this conversation I felt heard by the other.^[[5]](#footnote-5)^

- Disagree completely
- Disagree
- Disagree a bit
- Neutral
- Agree a bit
- Agree
- Agree completely

Think back to the online conversation you just described and indicate to what extent you agree with each of the following statements. Note: these statements are about YOUR experiences during the conversation.

In this conversation...

|  | Disagree completely | Disagree | Neutral | Agree | Agree completely |
| --- | --- | --- | --- | --- | --- |
| …I could say what I really wanted to say. |  |  |  |  |  |
| ... I could express my thoughts. |  |  |  |  |  |
| … I felt inhibited to say what I wanted to say. |  |  |  |  |  |
| ... the other was more concerned with him/herself than with what I said. |  |  |  |  |  |
| … the other listened to what I said. |  |  |  |  |  |
| … the other paid attention to what I said |  |  |  |  |  |
| ... the other tried to put him/herself in my shoes. |  |  |  |  |  |
| ... the other was insensitive to my thoughts and feelings. |  |  |  |  |  |
| ... the other was empathetic. |  |  |  |  |  |
| … the other showed genuine interest in me. |  |  |  |  |  |
| ... the other took me seriously. |  |  |  |  |  |
| ... the other treated me with respect. |  |  |  |  |  |
| … we looked at things differently. |  |  |  |  |  |
| ... we understood each other. |  |  |  |  |  |
| ... we were on the same wavelength. |  |  |  |  |  |
| ...now select "Disagree". |  |  |  |  |  |

Now here are some questions about the impact of COVID-19 (the coronavirus) on your daily life.

Please indicate how often you have experienced the following emotions in the past week.

|  | Never | Rarely | Sometimes | Often | Constantly |
| --- | --- | --- | --- | --- | --- |
| Anxious |  |  |  |  |  |
| Stressed |  |  |  |  |  |
| Sad |  |  |  |  |  |
| Lonely |  |  |  |  |  |

Since the corona crisis, how many different people do you speak to ONLINE per day on average?

- None
- 1
- 2
- 3
- More than 3

How many was that on average per day before that?

- None
- 1
- 2
- 3
- More than 3

Since the corona crisis, how many different people do you speak to ORALLY (i.e. offline or face-to-face) per day on average?

- None
- 1
- 2
- 3
- More than 3

How many was that on average per day before that?

- None
- 1
- 2
- 3
- More than 3

Since the corona crisis, how often do you leave the house on average?

- At least once a day
- At most once a day
- At most once every two days
- At most once every three days
- At most once a week

You are approaching the end of this study. If you have any comments, please leave them in the text box below (optional).

________________________________________________________________

________________________________________________________________

**1.3 Interviews Study 1^[[6]](#footnote-6)^**

To gain additional insight into the meaning of feeling heard, we conducted semi-structured interviews with four people that we expected to have practical experience with and knowledge about feeling heard. We asked what feeling heard meant to them, and about the situations, the preconditions and the consequences of (not) feeling heard.

The first interviewee (hereafter “I1”) was the former director of the Dutch compensation fund for violent crimes, who successfully changed the way her employees communicate with clients about procedures and outcomes. In effect, her intervention made clients feel more heard. I1 noticed that the money the fund gives to victims was seen as a sort of recognition. She further noticed that “*people wanted to tell their story, wanted to be heard*”. She therefore instructed her employees to “*listen to hear more about the story and also give something back [to show] that you really heard someone. Second is to explain the context and manage the expectations of what is possible and what isn’t.*” This had to be done in a clear and calm manner in colloquial speech. I1 further instructed her employees that, once a decision had been made, they had to explain this decision and give clients the opportunity to react, also to check whether the decision was based on correct information. This should signal that one takes the client and their story serious and that one puts in effort to help them. In this way, I1 attempted to remove the straitjacket imposed by the impersonal juridic procedures. She thinks that a lot of anger towards the government is instigated by being seen as a number. Thus, the personal encounter is of importance: “*That you are a governmental agency with a face or a voice*”. The interventions greatly improved client satisfaction. I1 quoted the feedback she got from clients as: “*I do not get any money, that’s unfortunate, but I am still very happy because you are the first person that wants to listen to me*”. From this she concluded that one can give recognition without providing what is asked for, so that the recognition can be in the procedure by clearly explaining why you make a decision. “*There can be disagreements about the content, but how do you find the form in which you can respectfully disagree?*” Importantly, also the employees putting the new approach into practice felt enriched by the positive responses it elicited.

The I1 was helped by a researcher that often acts as a mediator in conflicts between citizens and government; a context in which feeling heard also plays an important role. This person was our second interviewee (hereafter “I2”). She thinks that feeling heard becomes especially important in situations where people loose autonomy and are dependent on others, so where they have to deal with others making decisions for them that have an impact on their life. I2 believes that feeling heard in these situations often leads to more trust in the person making the decision. In her work, she relies on the procedural justice literature: “*That means that people feel heard during the process, and, in the way I do it, that virtually always goes hand in hand with personal contact. That means that people are able to tell their story, [and] that they notice that the story has landed with the person who listens*.” She stated that the expectations and goals of both parties have to be mentioned and recognized at the start of the conversation. When people’s interests clash, the recognition of that fact and of the potential frustrations that this yields, already helps a lot by bringing clarity. But she also stressed the importance of sensing whether the other person is ready to listen to your explanation, and not still in fight-flight-or-freeze modus, which is often forgotten in practice. I2 also referred to not feeling heard as an instigator for protest and polarization: “*Oftentimes when people do not feel heard, they start to look for support, and when they do not feel heard after several attempts, that can lead to frustration, conflict, and also expansion of the conflict, because people that do not feel heard will often seek support from others and that with that also an escalation […], or, at any rate, polarization can arise.*” According to I2, this would give rise to a strong us-versus-them rhetoric and make emotions run high.

Our third interviewee (hereafter “I3”) is a speech therapist who leads an organization that helps people overcome stuttering by means of speech therapy. He explained that people that stutter have difficulty expressing themselves and might therefore also feel less heard. He told us that this hearing applies on a more abstract level as well: the parents of children that stutter are often so focused on “curing” and “improving” the child that the child feels like a failure and not heard and not understood as a person, but treated as a number. This breeds anger and a lot of frustration, but also makes people who stutter feel guilty and lack self-esteem. According to I3, stuttering is a symptom of this underlying tension of feeling not good enough. He also mentioned an interesting reason for not feeling heard: “*The reason why a lot of people do not feel heard is because the listener is often self-involved*.” If someone is speaking, they immediately think: “*ok, what can I do with this, how should I react to this, how does this fit in my world-view?*” Then they are not completely open to what the other tries to convey. I3 observed that this also tends to happen a lot with people who stutter, because interaction partners focus on the stuttering itself and start to worry about how they can help, which distracts from what the person who stutters wants to say. He mentioned that it would help to stay calm and patient, and open to really listen to what the person tells you, surrendering to the moment and to the other. “*But it has to come from two sides, I think. The listener can be too self-involved, but also the speaker, which makes him or her not open to that connection and thus not felt heard*.” So, the bidirectional connection is important to feel heard. According to I3, simple attentive listening is not enough, it should be sincere: “*If someone is trying too hard, then I pull back a bit, I find that a bit weird, because someone is trying too hard, there is no need for that, why is that?*” Thus, being too attentive, can also raise the suspicion of ulterior motives. He thinks that feeling heard is strongly related to being on one line and feeling understood, which is of importance to the formation of a good and close relationship.

To gain some initial insight into the role of culture in feeling heard, our fourth interviewee (hereafter “I4”) was a Turkish-Dutch social scientist. He brought an interesting perspective by shifting the focus to cultural norms that dictate when someone has the right to speak. In the Turkish culture that he characterizes as patriarchic and quite authoritarian, feeling heard is influenced by “*whether or not one is allowed to have a voice at all in a certain context*”. I4 mentioned that this strongly depends on the topic of discussion and the role of the person in the community. There are certain themes about which not everyone can have a say. The right to speak is dependent on age in particular and also on gender. For example, in the case of a violation of the honor of a traditional family, only the elderly will gather to make a decision. It is even a Turkish saying: “*When the elderly speak the children remain silent*.” Similarly, I4 explained that there are male and female themes. The right to speak thus has to be earned by gaining status, making feeling heard status-dependent. This status can be earned by showing signs of adulthood, e.g., marriage, job. So, status confers the right to be heard, but also the other way around being heard confers status. The moment someone is allowed to take the floor and is not interrupted, this means that he or she is being listened to and thus has the right or status to speak. These rules of speaking and hearing are not written down but handed down by socialization. I4 thinks that if someone internalizes authoritarian values and therefore accepts hierarchy, this person might also accept not having a say about certain things. “*So, then it might also be acceptable to not be heard*”. But, according to I4, the need to be recognized as a person is part of human nature, which also involves the need to have a say about certain things, especially if it involves you.

**2.1 Tables & figure Study 2**

**Table 12. A characterization of the described conversations based on the percentage of participants scoring in each category of the conversation characteristics.**

| Question | Answer option | Percentage |
| --- | --- | --- |
| How many interaction partners? | One | 71.10% |
|  | Two | 13.10% |
|  | Three | 7.70% |
|  | More than three | 8.10% |
| Acquaintance before the conversation? | Very well | 59.60% |
|  | Quite well | 22.50% |
|  | A bit | 11.70% |
|  | Barely | 3.40% |
|  | Not at all | 2.80% |
| Feelings about relationship? | Very positive | 39.60% |
|  | Positive | 41.20% |
|  | Neutral | 12.90% |
|  | Negative | 4.50% |
|  | Very negative | 1.80% |
| Equal relationship? | Equal | 70.30% |
|  | Self authority | 5.40% |
|  | Other authority | 21.30% |
|  | Don’t know | 3.00% |

*Note.* *N* = 1000.


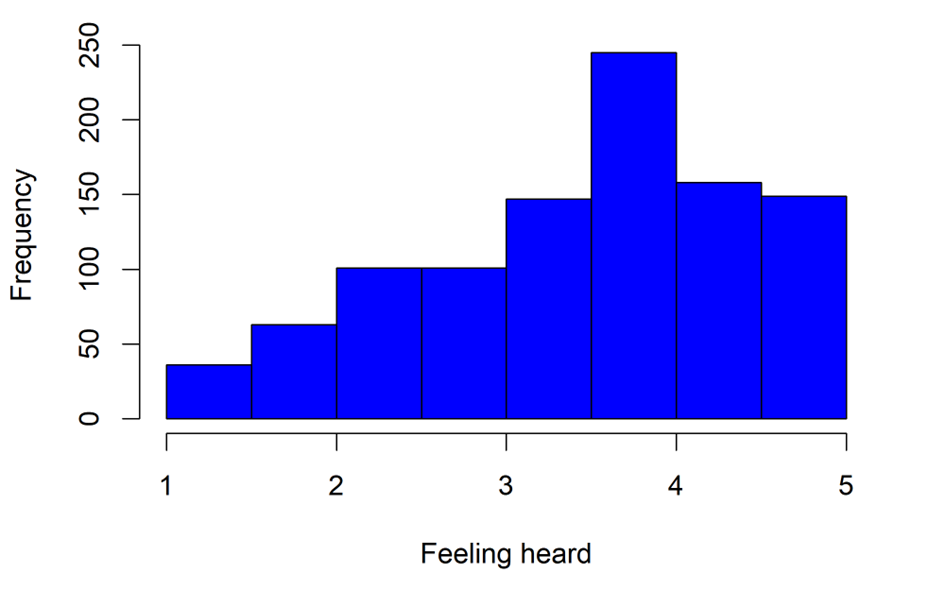


**Figure 2.** **The frequency distribution of feeling heard.**

**Table 13. The correlations between the feeling heard items as well as their respective means and standard deviations.**

| Item | Mean (SD) | 2 | | 3 | | | 4 | | | 5 | | | 6 | | 7 | | | | 8 | |  |
| --- | --- | --- | --- | --- | --- | --- | --- | --- | --- | --- | --- | --- | --- | --- | --- | --- | --- | --- | --- | --- | --- |
| 1. I Felt  Heard | 3.57 (1.23) | .62 | | -.62 | | | .79 | | | .65 | | | -.58 | | .73 | | | | .79 | |  |
| 2. I Speak Freely | 3.84 (1.19) |  | | -.45 | | | .61 | | | .49 | | | -.45 | | .59 | | | | .63 | |  |
| 3. Other Self Concerned | 2.92 (1.30) |  | |  | | | -.61 | | | -.51 | | | .66 | | -.57 | | | | -.60 | |  |
| 4. Other Listened | 3.74 (1.10) |  | |  | | |  | | | .65 | | | -.58 | | .73 | | | | .77 | |  |
| 5. Other Took Perspective | 2.88 (1.16) |  | |  | | |  | | |  | | | -.46 | | .60 | | | | .64 | |  |
| 6. Other Insensitive | 2.47 (1.29) |  | |  | | |  | | |  | | |  | | -.64 | | | | -.59 | |  |
| 7. Other Respectful | 3.84 (1.09) |  | | |  | | |  | | |  | | |  | | |  | .74 | | | |
| 8. We Understood | 3.60 (1.24) |  |  | | |  | | |  | | |  | | | |  | | | |  | |

*Note.* All the correlations reported in this table are significant at *** *p* < .001.

**Table 14. The results of the hierarchical regression on negative avoidance intentions.**

| Variable | Block 1 | | Block 2 | | Block 3 | | Block 4 | |
| --- | --- | --- | --- | --- | --- | --- | --- | --- |
|  | *b*  (SE) | *β* | *b*  (SE) | *β* | *b*  (SE) | *β* | *b*  (SE) | *β* |
| Age | -.01*  (.00) | -.07 | -.01*  (.00) | -.07 | -.00  (.00) | -.05 | -.00  (.00) | -.04 |
| Gender^a^  Man | .04  (.08) | .02 | .08  (.07) | .03 | .13*  (.06) | .06 | .11  (.06) | .05 |
| Gender Other | -.02  (.30) | -.00 | -.13  (.26) | -.01 | -.23  (.23) | -.02 | -.27  (.22) | -.03 |
| Education^b^  Middle | -.12  (.12) | -.04 | -.28**  (.10) | -.10 | -.18*  (.09) | -.06 | -.15  (.09) | -.05 |
| Education High | -.06  (.10) | -.02 | -.13  (.09) | -.05 | -.02  (.08) | -.01 | .02  (.07) | .01 |
| Employ-ment^c^  None | .19*  (.09) | .07 | .20*  (.08) | .07 | .21**  (.07) | .07 | .22**  (.07) | .08 |
| Employ-ment  Student | -.27*  (.13) | -.07 | -.22  (.11) | -.06 | -.22*  (.10) | -.06 | -.24*  (.09) | -.07 |
| Number of others |  |  | -.01  (.04) | -.01 | -.02  (.03) | -.02 | -.03  (.03) | -.02 |
| Know before |  |  | .17***  (.04) | .15 | .04  (.03) | .04 | .04  (.03) | .03 |
| Quality relation |  |  | -.61***  (.04) | -.48 | -.20***  (.04) | -.16 | -.05  (.05) | -.04 |
| Equality^d^  Other authority |  |  | .26**  (.08) | .09 | .18*  (.07) | .06 | .07  (.07) | .02 |
| Equality Self authority |  |  | -.20  (.15) | -.04 | -.30*  (.13) | -.06 | -.42***  (.13) | -.08 |
| Equality  Don’t know |  |  | .29  (.20) | .04 | .16  (.17) | .02 | .11  (.17) | .02 |
| Feeling heard |  |  |  |  | -.64***  (.04) | -.54 | -.29***  (.06) | -.24 |
| Intimacy |  |  |  |  |  |  | -.11*  (.05) | -.13 |
| Domin-ance |  |  |  |  |  |  | .12***  (.04) | .12 |
| Respons-iveness |  |  |  |  |  |  | .01  (.03) | .02 |
| Distrust |  |  |  |  |  |  | .05  (.04) | .06 |
| Attraction |  |  |  |  |  |  | -.06*  (.03) | -.11 |
| Goal accomp. |  |  |  |  |  |  | -.09*  (.03) | -.09 |
| Comm. appr. |  |  |  |  |  |  | .08**  (.03) | .07 |
| *R^2^* | .01 | | .23 | | .42 | | .46 | |
| *R^2^* adj. | .01 | | .22 | | .42 | | .45 | |
| *F* | 1.81  (df = 7; 992) | | 22.37***  (df = 13; 986) | | 51.71***  (df = 14; 985) | | 39.39***  (df = 21; 978) | |

*Notes.* **p* < .01; ***p* < .05; ****p* < .001

As the maximum values of the Generalized Variance Inflation Factor (GVIF) were 1.29 for Model 1, 1.38 for Model 2, 1.91 for Model 3, and 5.25 for Model 4, there was no reason to suspect problematic multicollinearity.

^a^ The reference level of gender was woman.

^b^ The reference level of education was low.

^c^ The reference level of employment status was working.

^d^ The reference level of equality of relationship was equal.

**Table 15. The results of the univariate linear regressions of the other behavioral intention measures on feeling heard, controlling for the effect of the covariates and the demographical variables.**

|  | *b* (SE) | *β* | *F*(1,998) | *R^2^* |
| --- | --- | --- | --- | --- |
| Positive avoidance intentions | -.57 (.04) | -.50 | 239.98*** | .29 |
| Negative approach intentions | -.22 (.04) | -.21 | 34.39*** | .11 |
| Positive approach intentions | .29 (.03) | .33 | 102.12*** | .28 |

*Note.* *** *p* < .001. We report the unadjusted multiple R^2^.

**2.2 Questionnaire Study 2^[[7]](#footnote-7)^**

Thank you for reading this information sheet and considering taking part in this research.

- Consent. Please check this box to indicate that you have read and understood the information on this form, are aged 18 or over, speak fluent English, and agree to take part in this survey.
- No consent. If you do NOT wish to take part in this study, please check this box.

Think back to a conversation in which you wanted to share your thoughts or feelings, make a point, speak your mind, and/or get something done (regardless of whether this was successful or not).
 
Describe this conversation in a couple of sentences below:
________________________________________________________________

________________________________________________________________

Besides you, how many people were there in this conversation?

- 1 other
- 2 others
- 3 others
- More than 3 others, please specify: _____

The following statements are about your experiences of the conversation you just described.
Please rate your agreement with each of these statements.

In this conversation, ...

|  | Strongly disagree | Disagree | Neutral | Agree | Strongly agree |
| --- | --- | --- | --- | --- | --- |
| ...I felt heard by the other person. |  |  |  |  |  |
| ...I could say what I really wanted to say. |  |  |  |  |  |
| ...the other person was more concerned with him/herself than with what I said. |  |  |  |  |  |
| ...the other person listened to what I said. |  |  |  |  |  |
| ...the other person tried to put him/herself in my shoes. |  |  |  |  |  |
| ...the other person was insensitive to my thoughts and feelings. |  |  |  |  |  |
| ...the other person treated me with respect. |  |  |  |  |  |
| ...we understood each other. |  |  |  |  |  |

The following statements are about your perception of the other person in the conversation you described.
Please rate your agreement with each of these statements.
 
The other person...

|  | Strongly disagree |  |  | Neutral |  |  | Strongly agree |
| --- | --- | --- | --- | --- | --- | --- | --- |
| ...was intensely involved in our conversation. |  |  |  |  |  |  |  |
| ...did not want a deeper relationship with me. |  |  |  |  |  |  |  |
| ...was not attracted to me. |  |  |  |  |  |  |  |
| ...seemed to find our conversation stimulating. |  |  |  |  |  |  |  |
| ...communicated coldness rather than warmth. |  |  |  |  |  |  |  |
| ...created a sense of distance between us. |  |  |  |  |  |  |  |
| ...acted bored by our conversation. |  |  |  |  |  |  |  |
| ...was interested in talking to me. |  |  |  |  |  |  |  |
| ...showed enthusiasm while talking to me. |  |  |  |  |  |  |  |
| ...made me feel he/she was similar to me. |  |  |  |  |  |  |  |
| ...tried to move the conversation to a deeper level. |  |  |  |  |  |  |  |
| ...acted like we were good friends. |  |  |  |  |  |  |  |
| ...seemed to desire further communication with me. |  |  |  |  |  |  |  |
| ...seemed to care if I liked him/her. |  |  |  |  |  |  |  |
| ...was sincere. |  |  |  |  |  |  |  |
| ...was interested in talking with me. |  |  |  |  |  |  |  |
| ...wanted me to trust him/her. |  |  |  |  |  |  |  |
| ...was willing to listen to me. |  |  |  |  |  |  |  |
| ...was open to my ideas. |  |  |  |  |  |  |  |
| ...was honest in communicating with me. |  |  |  |  |  |  |  |

The following statements are about your perception of the other person in the conversation you described.
Please rate your agreement with each of these statements.
 
The other person...

|  | Strongly disagree |  |  | Neutral |  |  | Strongly agree |
| --- | --- | --- | --- | --- | --- | --- | --- |
| ...attempted to persuade me. |  |  |  |  |  |  |  |
| ...did not attempt to influence me. |  |  |  |  |  |  |  |
| ...tried to control the interaction. |  |  |  |  |  |  |  |
| ...tried to gain my approval. |  |  |  |  |  |  |  |
| ...didn’t try to win my favor. |  |  |  |  |  |  |  |
| ...had the upper hand in the conversation. |  |  |  |  |  |  |  |
| ...considered us equals. |  |  |  |  |  |  |  |
| ...It’s important that you pay attention to this study. Please select “Strongly disagree”. |  |  |  |  |  |  |  |
| ...did not treat me as an equal. |  |  |  |  |  |  |  |
| ...wanted to cooperate. |  |  |  |  |  |  |  |

Please indicate to what extent you think the following characteristics apply to the other person in the conversation you described.

|  | 1 | 2 | 3 | 4 | 5 | 6 | 7 |  |
| --- | --- | --- | --- | --- | --- | --- | --- | --- |
| Trustworthy |  |  |  |  |  |  |  | Untrustworthy |
| Trustful of this person |  |  |  |  |  |  |  | Distrustful of this person |
| Confidential |  |  |  |  |  |  |  | Divulging |
| Benevolent |  |  |  |  |  |  |  | Exploitive |
| Safe |  |  |  |  |  |  |  | Dangerous |
| Candid |  |  |  |  |  |  |  | Deceptive |
| Not Deceitful |  |  |  |  |  |  |  | Deceitful |
| Straightforward |  |  |  |  |  |  |  | Tricky |
| Respectful |  |  |  |  |  |  |  | Disrespectful |
| Considerate |  |  |  |  |  |  |  | Inconsiderate |
| Honest |  |  |  |  |  |  |  | Dishonest |
| Reliable |  |  |  |  |  |  |  | Unreliable |
| Faithful |  |  |  |  |  |  |  | Unfaithful |
| Sincere |  |  |  |  |  |  |  | Insincere |
| Careful |  |  |  |  |  |  |  | Careless |

The following questions are about the way you feel towards the other person in the conversation you described.

How unpleasant/pleasant do you feel about the other person?

- Extremely unpleasant
- Extremely pleasant

How cold/warm do you feel about the other person?

- Extremely cold
- Extremely warm

How positive/negative do you feel about the other person?

- Extremely positive
- Extremely negative

How distant/close do you feel to the other person?

- Extremely distant
- Extremely close

How friendly/unfriendly do you feel toward the other person?

- Extremely friendly
- Extremely unfriendly

Please answer the following questions about the other person in the conversation you described.^[[8]](#footnote-8)^
  
The other person...

|  | Not at all true |  | Some-what true |  | Mode-rately true |  | Very true |  | Compl-etely true |
| --- | --- | --- | --- | --- | --- | --- | --- | --- | --- |
| ...really listens to me. |  |  |  |  |  |  |  |  |  |
| ...is responsive to my needs. |  |  |  |  |  |  |  |  |  |
| ...is an excellent judge of my character. |  |  |  |  |  |  |  |  |  |
| ...sees the “real” me. |  |  |  |  |  |  |  |  |  |
| ...sees the same virtues and faults in me as I see in myself. |  |  |  |  |  |  |  |  |  |
| ...“gets the facts right” about me. |  |  |  |  |  |  |  |  |  |
| ...is aware of what I am thinking and feeling. |  |  |  |  |  |  |  |  |  |
| ...understands me. |  |  |  |  |  |  |  |  |  |
| ...is on “the same wavelength” with me. |  |  |  |  |  |  |  |  |  |
| ...knows me well. |  |  |  |  |  |  |  |  |  |
| ...esteems me, shortcomings and all. |  |  |  |  |  |  |  |  |  |
| ...values and respects the whole package that is the “real” me. |  |  |  |  |  |  |  |  |  |
| ...seems to focus on the “best side” of me. |  |  |  |  |  |  |  |  |  |
| ...expresses liking and encouragement for me. |  |  |  |  |  |  |  |  |  |
| ...seems interested in what I am thinking and feeling. |  |  |  |  |  |  |  |  |  |
| ...seems interested in doing things with me. |  |  |  |  |  |  |  |  |  |
| ...values my abilities and opinions. |  |  |  |  |  |  |  |  |  |
| ...respects me. |  |  |  |  |  |  |  |  |  |

The following statements are about the extent to which you accomplished your goals in the conversation you described.
Please rate your agreement with each of these statements.

|  | Strongly disagree | Disagree | Neutral | Agree | Strongly agree |
| --- | --- | --- | --- | --- | --- |
| In this conversation, I accomplished the goals I had prior to this conversation. |  |  |  |  |  |
| I was unsuccessful in achieving what I wanted in this conversation. |  |  |  |  |  |
| I got what I aimed for in this conversation. |  |  |  |  |  |

The following statements are about the way you **generally** feel about communicating with other people.
Please indicate the degree to which each statement applies to you.

|  | Strongly disagree | Disagree | Undecided | Agree | Strongly agree |
| --- | --- | --- | --- | --- | --- |
| While participating in a conversation with a new acquaintance, I feel very nervous. |  |  |  |  |  |
| I have no fear of speaking up in conversations. |  |  |  |  |  |
| Ordinarily I am very tense and nervous in conversations. |  |  |  |  |  |
| It’s important that you pay attention to this study. Please select “Undecided”. |  |  |  |  |  |
| Ordinarily I am very calm and relaxed in conversations. |  |  |  |  |  |
| While conversing with a new acquaintance, I feel very relaxed. |  |  |  |  |  |
| I’m afraid to speak up in conversations. |  |  |  |  |  |

Now, think back again to the conversation you described at the start of this questionnaire.
What would you do if you were to have another conversation with this same person?
Please indicate to what extent you agree with each of the following statements.

Next time, …

|  | Strongly disagree | Disagree | Neutral | Agree | Strongly agree |
| --- | --- | --- | --- | --- | --- |
| …I would avoid having this conversation altogether. |  |  |  |  |  |
| …I would withdraw myself from this conversation. |  |  |  |  |  |
| …I would hold back in conversation. |  |  |  |  |  |
| …I would change the topic compared to the last conversation. |  |  |  |  |  |
| …when discussing the same topic, I would make my point(s) more forcefully. |  |  |  |  |  |
| …I would stand my ground. |  |  |  |  |  |
| …I would enter the conversation with an open mind. |  |  |  |  |  |
| ...I would be as nice as possible to the other person. |  |  |  |  |  |

The following questions are about your relationship with the other person **before** you had the conversation you described.

 How well did you know this person before the start of the conversation?

- Very well
- Quite well
- A bit
- Barely
- Not at all

In general, how do you feel about your relationship with this person?

- Very positive
- Positive
- Neutral
- Negative
- Very negative

In general, do you consider yourself to be on an equal footing with this person?

- Yes, we are equals
- No, the other has more authority (e.g., the other is my employer)
- No, I have more authority (e.g., the other is my child)
- Don't know

To conclude this survey, we want to ask you a couple of demographic questions.

How old are you?

*Dropdown ranging from “Under 18” to “100”*

What gender do you currently identify with?

- Woman
- Man
- Other
- Prefer not to say

Which country do you live in?

- United States
- England
- Other, please specify: _____

What is the highest education level you have completed?

- No formal qualifications
- Secondary school/GCSE
- College/A levels
- Undergraduate degree (BA/BSc/other)
- Graduate degree (MA/MSc/MPhil/other)
- Doctorate degree (PhD/MD/other)

Q25.6 What is your current employment status?

- Employed full-time
- Employed part-time
- Self-employed
- Unemployed
- Retired
- Student
- Other, please specify: _____

Q26.1 You are approaching the end of this survey. If you have anything to add, please do so in the text box below (optional).

________________________________________________________________

________________________________________________________________

**2.3 Reasoning behind the scales included to assess validity**

To establish convergent and divergent validity, we searched the literature for measures that 1) relate to the construct of feeling heard, without being an aspect of feeling heard (e.g., empathy, respect), and 2) are relevant in the context of interpersonal interactions. Interestingly, we found very few scales measuring conversational experiences that do not measure aspects of feeling heard. Most related scales measure general perceptions and feelings towards either the self, partner(s), or relationship, rather than the experiences and perceptions of the interaction partner(s) within a single conversation. A notable exception is the relational communication scale (Burgoon & Hale, 1987). But, in contrast to feeling heard, this scale only taps into the perceived behavior of the listener and thus is measured exclusively on the “you-level”. Nevertheless, we deemed the scale’s sub-scales of conversational intimacy and dominance vs. equality very relevant for feeling heard experiences.

We further thought feeling heard might be intertwined with other positive feelings towards interaction partner(s), most prominently trust and liking. We therefore included the established measure of individualized (dis)trust (Wheeless & Grotz, 1977), and an indicator of interpersonal liking that best fitted the context: the affective attraction scale (Montoya & Insko, 2008).

As mentioned in the introduction, perceived partner responsiveness (Reis et al., 2018) might be very similar to feeling heard. Since the perceived partner responsiveness scale is designed to assess people’s perceptions of their relationship partner’s behaviors and attitudes towards them in general, items include the word “usually”. This word was removed to better fit the current context, because presumably not all described conversations involved familiar others.

The content analysis of Study 1 suggested that perceiving effort on the side of the listener might be a defining feature of feeling heard. This essentially came down to the accomplishment of conversation goals, e.g., getting things done, receiving answers. As we could not find a suitable and validated measure of conversational goal accomplishment, we designed a reliable three-item scale to assess this.

Lastly, feeling heard might be closely connected to communication anxiety. People that do not feel heard might simply feel uncomfortable in conversation. We only included the dyad subscale of the communication apprehension scale (McCroskey, 1982), since the other subscales are not applicable to all conversation contexts that participants could potentially come up with.

**3.1 Study 3^[[9]](#footnote-9)^**

Having developed and validated the scale, Study 3 tests whether the experience of feeling heard can be experimentally manipulated. Research suggests that text-based online communication can lead people to feel not heard (Roos et al., 2020a). We therefore tried to manipulate feeling heard by asking people to engage in two forms of mediated communication: through text and through audio channels.

**Aims and hypotheses**

We expected that participants would feel more heard when they were able to communicate by voice than when they were restricted to communicating by text (Hypothesis 1). We further predicted feeling heard to correlate positively with, but also to still be distinct from, other relevant variables: solidarity, conversational flow, and self-esteem (Hypothesis 2). We also explored the effects of different roles (sender vs. responder) within media conditions. The latter was aimed to investigate how the role by condition interaction effect on feeling heard compared to the other variables. This study’s design was preregistered prior to data collection at <https://osf.io/bkd7t/?view_only=a3cad5dc34384c74bb3ec99ac954c6cf>. As can be seen in the preregistration, the original purpose and hypotheses of this study differ from the ones reported in this paper. There was one more condition in the original study: the VoxBox condition where participants could use a keyboard to make interjecting sounds, like “hmhm” and “yes”. We ignore this third condition here because we are only interested in the differences between verbal and textual communication.

**Method**

**Participants and post-hoc power**

The original sample consisted of 78 students that participated for partial course credit, but three dyads were discarded because they either did not follow the instructions or did not finish the experiment. In the final sample (*N* = 74; *M_age_* = 20.18, *SD_age_* = 2.24; 66.22% female, 31.08% male, 2.70% other) there were 72 first-year psychology students. English fluency was an inclusion criterion. The experiment involved dyads, 91.89% of whom did not know or barely knew each other before the experiment started.

A sensitivity analysis showed that this sample size yielded 80% power to detect between-condition (within-subjects) effects of at least *F* = .17 (small effect; Cohen, 1988) with alpha .05 (two-tailed), assuming sphericity and a correlation of .50 among repeated measures. Similarly, within-between interaction effects of at least *F* = .17 could be detected with 80% power, and alpha .05 (two-tailed).

**Design and procedure**

Participants entered the lab in duos. Both participants were immediately seated in separate cubicles with a computer with a headset. The study had a 2x2 factorial design with a communication medium condition: talking vs. typing, and a participant role condition: sender vs. responder. Communication medium was a repeated measures factor on the dyad level: each dyad engaged in the talking and the typing condition (in randomized order). Participant role was a between-participants factor and nested within dyads: each participant was either sender or responder (randomly allocated) and kept this role throughout both conditions.

In both conditions, the sender told the responder about either their holidays, hobbies, or favorite TV shows through a headset. It is important to note that the sender always used audio, which means that the conditions were defined by the task of the responder. In the talking condition, the responder could talk back via a headset connected to the same audio channel that the sender used. In the typing condition, the responder could only react through text-based chat, which appeared on the screen of the sender. There was no visual contact in both conditions. The experimenter stopped the conversation after 5 minutes unless participants decided to finish beforehand. After each conversation, participants individually completed a questionnaire on their computers (see supplementary materials, section 3.2).^[[10]](#footnote-10)^

**Materials**

The feeling heard scale was still under development at the time of the study because it was conducted simultaneously with Study 1. Therefore, only four items of the feeling heard scale were included in the questionnaire: 1) “During this conversation, I felt heard by the other person”, 2) “During this conversation, I could express myself freely”, 3) “During this conversation, the other person respected what I [said/ wrote]”, and 4) “During this conversation, we misunderstood each other” (reverse coded). In Study 1, this four-item subscale correlated very strongly with the full feeling heard scale: *r* = .97. It thus captures 94% of the variance in the full scale. This means that we can assume that the selected items represent the full scale well. In the current study, the four items also formed a reliable scale with a hierarchical omega of .71.

We additionally measured three constructs that might be closely related to feeling heard. All items were measured on Likert scales ranging from 1 = *Strongly disagree* to 5 = *Strongly agree*. First, we measured solidarity with four items adapted from Koudenburg et al., (2015), for example, “During this conversation, I identified with the other person” and “During this conversation, I felt a sense of belonging with the other person” (ω = .80). Secondly, we measured conversational flow (Koudenburg et al., 2017) with four items: “This conversation was [coordinated and smooth/ difficult (reverse coded)/ pleasant/ harmonious]” (ω = .88). Lastly, we adjusted the Rosenberg (1965) self-esteem scale to construct a reliable three-item measure of conversational self-esteem: “During this conversation, I felt confident about myself”, “During this conversation, I experienced a sense of failure” (reverse coded), and “Right now, I feel good about myself” (ω = .80).

**Results**

We tested the main effect of medium condition (talking vs. typing), the main effect of participant role (sender vs. responder), and their interaction effect on feeling heard, solidarity, flow, and self-esteem. As participants were part of a dyad and were measured two times (in both conditions), the statistical analysis had to take into account these two sources of non-independence of observations. We therefore analyzed the data with multilevel repeated measures models, using the lmer function in the R package lme4 (version 1.1-21, Bates et al., 2019). For each dependent variable, we defined models with condition, role, and their interaction term as fixed-effect predictors, and participant and group as random effects. We used the emmeans package (version 1.5.5-1, Length et al., 2021) to estimate means and confidence intervals. These results are presented in Figure 1.

**Figure 1. The main and interaction effects of communication medium condition and participant role on the four dependent variables.**


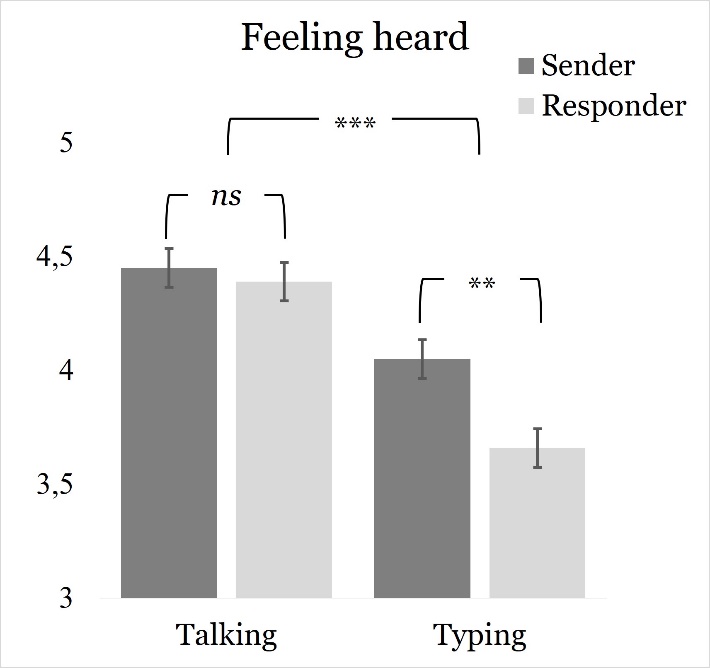

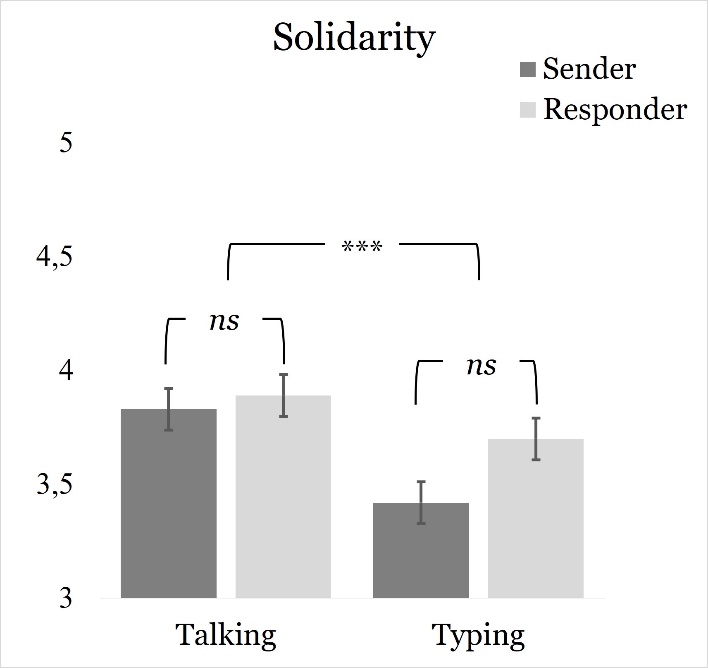

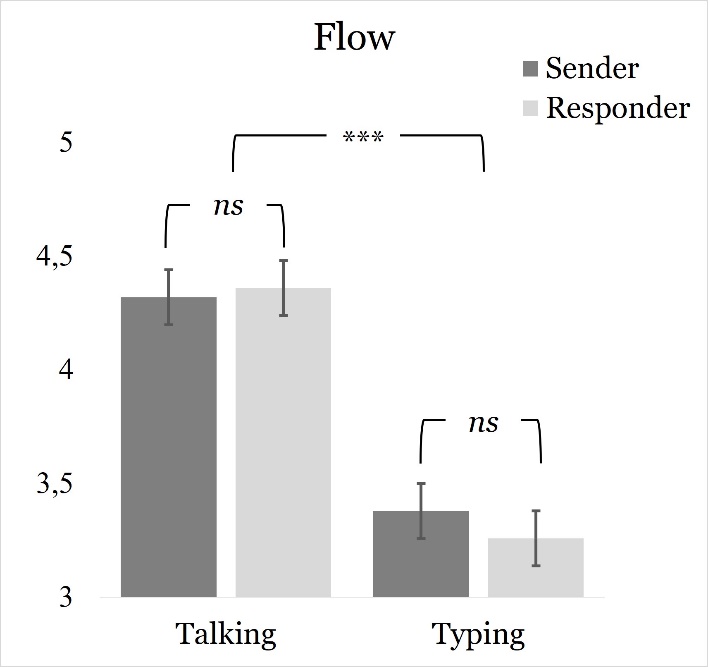

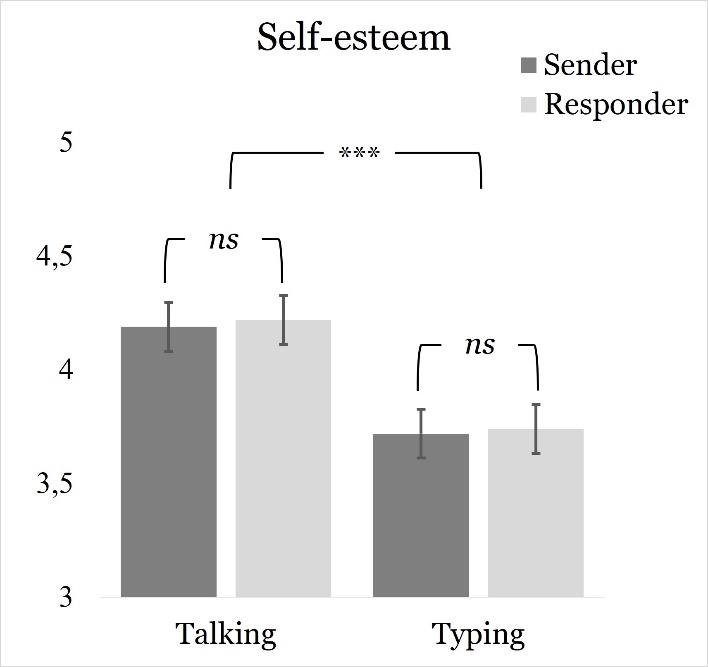


*Note.* 95% confidence intervals are given. *p* > .05 *^ns^*; *p* < .01 **; *p* < .001 ***

In line with Hypothesis 1, both senders and responders felt significantly less heard in the typing condition than in the talking condition (*F*(1,72) = 56.42, *p* < .001). They also felt less solidarity towards one another (*F*(1,72) = 15.14, *p* < .001), experienced their conversation as flowing less smoothly (*F*(1,72) = 83.08, *p* < .001), and felt less self-confident (*F*(1,72) = 24.27, *p* < .001) in the typing condition.

But importantly, feeling heard was the only variable that also showed a significant main effect for participant role (*F*(1,72) = 5.97, *p* = .017): responders felt less heard. This main effect was qualified by a significant role by condition interaction effect (*F*(1,72) = 5.26, *p* = .024). The simple main effects revealed no difference in feeling heard between senders and responders in the talking condition, but in the typing condition, responders felt significantly less heard than senders. For solidarity, flow, and self-esteem there was no main effect of participant role (*F*(1,72) = 2.90, *p* = .097, *F*(1,72) = 0.12, *p* = .730, and *F*(1,72) = 0.04, *p* = .835, respectively) and no role by condition interaction effect (*F*(1,72) = 2.26, *p* = .137, *F*(1,72) = 0.48, *p* = .491, and *F*(1,72) = 0.00, *p* = .963, respectively). Thus, the feeling heard measure picked up on additional variance of the differences manipulated in this study.

The distinct character of feeling heard is also evident in the partial correlations among the variables, see Table 1. In line with Hypothesis 2, while all variables were significantly positively correlated to feeling heard, there were no indications for redundancy because all correlations were below .80 (the highest is *r* = .65). Together with the condition effects, this shows that feeling heard is closely related to but also distinct from solidarity, conversational flow, and self-esteem.

**Table 1. Correlations between the four dependent variables (partialling out the effect of condition).**

| Variable | Feeling Heard | Solidarity | Flow |
| --- | --- | --- | --- |
| Solidarity | .33** |  |  |
| Flow | .65*** | .43*** |  |
| Self-esteem | .56*** | .38*** | .60*** |

Note. These are partial correlations between the variables, controlling for the effects of condition, calculated by taking the average of the correlations between all sets of variables in the two conditions (using z-transformations). *p* < .01 **; *p* < .001 ***

**Discussion**

The results of this study demonstrate that the FHS explains variance in conversational experiences that other variables do not. Although both parties felt less heard in the typing condition, responders were more affected than senders. Responders were clearly most limited in their communication (only by text), and likely also felt that their partner, who used voice and therefore could talk on while they were still typing, did not really listen and react well to them. Notably, none of the other variables picked up this differing experience between interaction partners. Both parties experienced equal amounts of solidarity, flow, and self-esteem, which were all reduced in the typing condition compared to the speaking condition. This finding shows not only that the FHS is highly sensitive to picking up differences in communication setting, but also that it explains unique variance that other variables do not.

Further, in line with previous research (Roos et al., 2020a), the current study showed that interaction partners felt less heard when they communicated via text-based chats than via audio-channels. In the typing condition, one of the interaction partners (the sender) spoke via a headset while the other (the responder) reacted via text messages rather than audio. This way, our design allowed us to partial out the unique effects of speaking via vs. listening to text-based communication. It appeared that both text-based speaking and listening led to reductions in feeling heard compared to the condition where both parties could speak. This is rather unsurprising for responders as the manipulation clearly restricted their voice. The fact that senders also felt less heard is less obvious. This can be explained by considering that responders could not use many of the passive engagement behaviors that make people feel heard (see open answers Study 1), since these are more difficult to enact in text-based chats. For example, listening is importantly communicated with short expressions like “hmm”, “yes”, and nodding (Reis & Clark, 2013). In contrast, speaker interruptions are more likely online. Senders might have interpreted all this unresponsiveness as signs of lacking attention, empathic concern, and respect on the side of responders. On top of this, both senders and responders likely experienced a reduced sense of “us” (also visible in their reduced solidarity ratings). This could be due to the time lags caused by the longer time typing takes, and its mismatch with the speed of talking, which disrupts the smooth conversational turn-taking that is needed for grounding (Koudenburg et al., 2013; Roos et al., 2020b).

**3.2 Questionnaire Study 3**

Permission to participate:

- Yes, I agree to participate in this study; this permission is valid until 07-03-2020.
- No, I do not agree to participate in this study.

Permission to process personal data:

- Yes, I consent to the processing of my personal data as mentioned in the research information. I know I can ask for my data to be deleted until 07-03-2022. I can also ask for this if I decide to stop participating in this study.
- No, I do not consent to the processing of my personal data.

Please have a conversation about [*your/the other person’s*] [*holidays/hobbies/favorite TV shows*]*.*

[*In the following conversation you and the other person can talk and listen to each other by using the headsets you are provided with./*

*In the following conversation you can communicate with the other person by typing on the keyboard. You will hear your communication partner via the headset./*

*In the following conversation you can communicate with the other person by typing on the keyboard. You will hear your communication partner via the headset. Moreover, you are equipped with a device that can make vocalization sounds as you would use them during a common personal conversation. To understand what the device does, you will get a minute to test it out./*

*In the following conversation you can talk to the other person through the headset. You can read your communication partner's input to the conversation on your screen./*

*In the following conversation you can talk to the other person through the headset. You can read your communication partner's input to the conversation on your screen. Further your conversation partner can make interjecting sounds with help of an additional keyboard.*]**^^[[11]](#footnote-11)^^**

Please signal to the researcher when you are ready to start by opening your door. You will be given further instructions.

You just had a talk about *your/the other person’s holidays/hobbies/favorite TV shows*. The following questions are about your personal impression and feelings about this conversation. Please answer them truthfully.

Please select for each statement how strong you agree or disagree with it.

This conversation was...

|  | Strongly disagree | Disagree | Neither agree nor disagree | Agree | Strongly agree |
| --- | --- | --- | --- | --- | --- |
| ...good. |  |  |  |  |  |
| ...smooth. |  |  |  |  |  |
| ...difficult. |  |  |  |  |  |
| ...pleasant. |  |  |  |  |  |
| ...harmonious. |  |  |  |  |  |

During this conversation...

|  | Strongly disagree | Disagree | Neither agree nor disagree | Agree | Strongly agree |
| --- | --- | --- | --- | --- | --- |
| ...I liked the other person. |  |  |  |  |  |
| ...I felt a sense of belonging with the other person. |  |  |  |  |  |
| ...I experienced a sense of unity with the other person. |  |  |  |  |  |
| ...I identified with the other person. |  |  |  |  |  |
| ...we misunderstood each other. |  |  |  |  |  |
| ...we were on the same wavelength. |  |  |  |  |  |

During this conversation...

|  | Strongly disagree | Disagree | Neither agree nor disagree | Agree | Strongly agree |
| --- | --- | --- | --- | --- | --- |
| ...I felt the other person likes me. |  |  |  |  |  |
| ...I felt accepted by the other person. |  |  |  |  |  |
| ...I felt heard by the other person. |  |  |  |  |  |
| ...I felt confident about myself. |  |  |  |  |  |
| ...I experienced a sense of failure. |  |  |  |  |  |
| Right now, I feel good about myself. |  |  |  |  |  |

During this conversation...

|  | Strongly disagree | Disagree | Neither agree nor disagree | Agree | Strongly agree |
| --- | --- | --- | --- | --- | --- |
| ...I could express myself freely. |  |  |  |  |  |
| ...I felt like my messages ‘came across’ to the other person. |  |  |  |  |  |
| ...the other person took my message into account. |  |  |  |  |  |
| ...the other person valued my input to the conversation. |  |  |  |  |  |
| ...the other person took me seriously. |  |  |  |  |  |
| ...the other person respected what I said/ wrote. |  |  |  |  |  |
| ...I envisioned my communication partner when talking to him/ her. |  |  |  |  |  |
| ...I felt like I dealt with a very real person. |  |  |  |  |  |
| ...I felt like talking to an abstract anonymous person. |  |  |  |  |  |

During this conversation, the other person...

|  | Strongly disagree | Disagree | Neither agree nor disagree | Agree | Strongly agree |
| --- | --- | --- | --- | --- | --- |
| ...was considerate. |  |  |  |  |  |
| ...expressed him/herself clearly. |  |  |  |  |  |
| ...expressed him/herself vaguely. |  |  |  |  |  |
| ...thought carefully about how he/she expressed him/herself. |  |  |  |  |  |
| ...expressed him/herself politely. |  |  |  |  |  |
| ...considered the consequences of what he/she was saying. |  |  |  |  |  |

Now we want to ask you some general questions to gain insight into the demographics of our participants.

Please indicate your age. _____

Please indicate your gender.

- Male
- Female
- Other

Are you a first year Psychology student?

- Yes, English bachelor
- Yes, Dutch bachelor
- No

What do you do in daily life?

Studying, namely: _____

Working, namely: _____

Please indicate how comfortable you are in speaking English?

- Very comfortable
- A bit comfortable
- Neutral
- A bit uncomfortable
- Very uncomfortable

What is your mother tongue? _____

Please indicate how well you knew the other participant before the experiment started.

- Very well
- A bit
- Barely
- Not at all

What differences did you notice between the three conversations you just had?

________________________________________________________________

________________________________________________________________

What did you think about the 'VocBox' (the button box that made vocalization sounds)?

________________________________________________________________

________________________________________________________________

Please indicate below briefly what you think the manipulation and aim of this study was.

________________________________________________________________

________________________________________________________________

Did you alter your behavior in any way to achieve a certain outcome regarding this aim. If yes, please indicate below how.

________________________________________________________________

________________________________________________________________

**4 The Feeling Heard Scale user manual**

The Feeling Heard Scale (FHS) was developed to measure feeling heard in everyday conversations. It is a concise eight-item scale with good psychometric properties. The FHS explains conversational experiences of consequence for social relationships and is a distinct and powerful predictor of future conversation intentions across different contexts and relationships.

We recommend using the following instructions for participants: “The following statements are about your experiences of [conversation]. Please rate your agreement with each of these statements.” The phrase [conversation] can be changed so it refers to a specific conversation or meeting that fits your research context.

The full version of the Feeling Heard Scale includes the following items (in fixed order):

In this conversation, ...

...I felt heard by the other person.

...I could say what I really wanted to say.

...the other person was more concerned with him/herself than with what I said.

...the other person listened to what I said.

...the other person tried to put him/herself in my shoes.

...the other person was insensitive to my thoughts and feelings.

...the other person treated me with respect.

...we understood each other.

We recommend recording agreement with these items on a five-point Likert-type scale ranging from 1 = *Strongly disagree* to 5 = *Strongly agree*. Other scales or a binary format (yes/no) have not been tested.

The items above are phrased in singular form but can easily be translated to plural form when meant to refer to multiple interaction partners by replacing “the other person” by “the other people” and conjugate verbs accordingly.

All items should be preceded by the phrase: “In this conversation” or “In this meeting”. We suggest doing this in the format as presented above.

To calculate the final feeling heard score, items 3 and 6 need to be reverse coded. After recoding, the individual item scores can be averaged. All items can be given the same weight.

The single item alternative to the full scale is: “In this conversation, I felt heard by the other person.” Using this single item implies losing predictive and explanatory power (see accompanying paper for the details). This loss of precision should be reported when using the single item. We recommend using it only in large samples and/or when little precision is required and/or when space does not permit the use of the full scale.

Anyone is free to use this scale as long as it is properly referenced (MASKED FOR REVIEW).

Please be aware that the FHS is still awaiting validation and standardization in clinical and other applied contexts. Nor is it validated for use with children or people with a non-western cultural background.

Items in Dutch:

In dit gesprek…

…voelde ik mij gehoord.

…heb ik kunnen zeggen wat ik graag wilde zeggen.

...was de ander meer met zichzelf bezig dan met wat ik zei.

…luisterde de ander naar wat ik zei.

...probeerde de ander zich in mij te verplaatsen.

…was de ander ongevoelig voor mijn gedachten en gevoelens.

…behandelde de ander mij met respect.

...begrepen we elkaar.

1. Translated to English by the first author. The original questionnaire was in Dutch. [↑](#footnote-ref-1)
2. We show the questionnaire in singular form (i.e., “the other), some of the items were rephrased to plural form (i.e., “the others”) when participants described a conversation with more than one other person. [↑](#footnote-ref-2)
3. The answer options below were not translated to English as they represent the Dutch education system which is rather different from that in the UK or US. [↑](#footnote-ref-3)
4. Participants saw only one of the italicized phrases, depending on conditions. [↑](#footnote-ref-4)
5. Due to a programming error, this single item was measured on a 7-point Likert scale. To make it compatible with the other items, it was rescaled to a 5-point scale before analysis. [↑](#footnote-ref-5)
6. The interviews were conducted in Dutch and audio-recorded by the first author. The English summary of these recordings is printed below. [↑](#footnote-ref-6)
7. We show the questionnaire in singular form. [↑](#footnote-ref-7)
8. In line with the original scale (Perceived Partner Responsiveness Scale; Reis et al., 2018), these items were presented in randomized order. [↑](#footnote-ref-8)
9. Part of this Study has been published before in a paper with a different approach and aim (Roos et al., 2022). [↑](#footnote-ref-9)
10. The questionnaire included two additional constructs that were not deemed of relevance to the purposes of the current paper: politeness and social presence. [↑](#footnote-ref-10)
11. Participants saw only one of the italicized phrases, depending on conditions. [↑](#footnote-ref-11)
